# Primary mucinous carcinoma with rhabdoid cells of the thyroid gland: a case report

**DOI:** 10.1186/s13000-016-0500-8

**Published:** 2016-06-10

**Authors:** Mioko Matsuo, Masazumi Tuneyoshi, Mari Mine

**Affiliations:** Department of Head and Neck Surgery, Japan Community Health Care Organization Kyushu Hospital, 1-8-1 Kishinoura, Yahatanishi-ku, Kitakyushu-City, Fukuoka 806-8501 Japan; Department of Anatomic Pathology, Pathological Sciences, Graduate School of Medical Sciences, Kyushu University, Fukuoka, Japan; Department of Pathology, Kyushu Center Hospital of the Mutual Aid Association of Public School Teachers, Fukuoka, Japan

**Keywords:** Thyroid cancer, Mucinous carcinoma, Rhabdoid cells, TSH suppression

## Abstract

**Background:**

Primary mucinous carcinoma of the thyroid gland is a rare disease; only 6 cases of primary mucinous carcinoma of the thyroid have been previously reported. Primary mucinous carcinoma of the thyroid gland with incomplete tumor resection tends to be associated with a poor prognosis, resulting in death within a few months. An early and appropriate diagnosis may contribute to improvement in patient prognosis; however, it is extremely difficult to diagnose primary mucinous carcinoma of the thyroid. We present the seventh reported case of primary mucinous carcinoma in the thyroid gland; moreover, rhabdoid cells were detected, which, to our knowledge, is a novel finding.

**Case presentation:**

An 81-year-old Japanese woman was initially diagnosed with a poorly differentiated thyroid carcinoma, and she underwent a hemithyroidectomy. Pathological examination revealed the presence of abundant mucus and agglomeration of large atypical cells. Rhabdoid cells were also seen scattered among the tumor cells. Immunostaining was performed for various markers, and on the basis of these results, we diagnosed the lesion as primary mucinous carcinoma with rhabdoid cells in the thyroid gland. Ten months after surgery, recurrence was noted in the paratracheal lymph nodes; therefore, total resection of the residual thyroid gland and paratracheal lymphadenectomy with thyroid-stimulating hormone suppression were performed. The patient is currently alive and disease-free.

**Conclusions:**

The current case is of interest not only because of the rare histological findings, but also because the patient achieved long-term survival following diagnosis of a mucinous carcinoma. We believe this report will be helpful for diagnosing future cases of mucinous carcinoma of the thyroid.

## Background

Mucinous carcinomas are characterized by an abundant extracellular production of mucus. They are usually detected in the breast, colon, stomach, and pancreas. At these sites, incidence rates range from 1 % to 5 % [1, 2]. Primary mucinous carcinomas of the thyroid gland are extremely rare. Since the first report published by Diaz et al. in 1976, only 6 such case reports were identified during our search of the PubMed database [3–8]. Furthermore, primary mucinous carcinoma of the thyroid gland with rhabdoid cells has never been previously reported. Here, we describe our experience with the first reported case of primary mucinous carcinoma of the thyroid gland with rhabdoid cells and discuss our findings in the context of the extant literature.

## Case presentation

An 81-year-old woman presented with a mass that had grown rapidly over a few days at the Kyushu Central Hospital of the Mutual Aid Association of Public School Teachers.

**Fig. 1:**
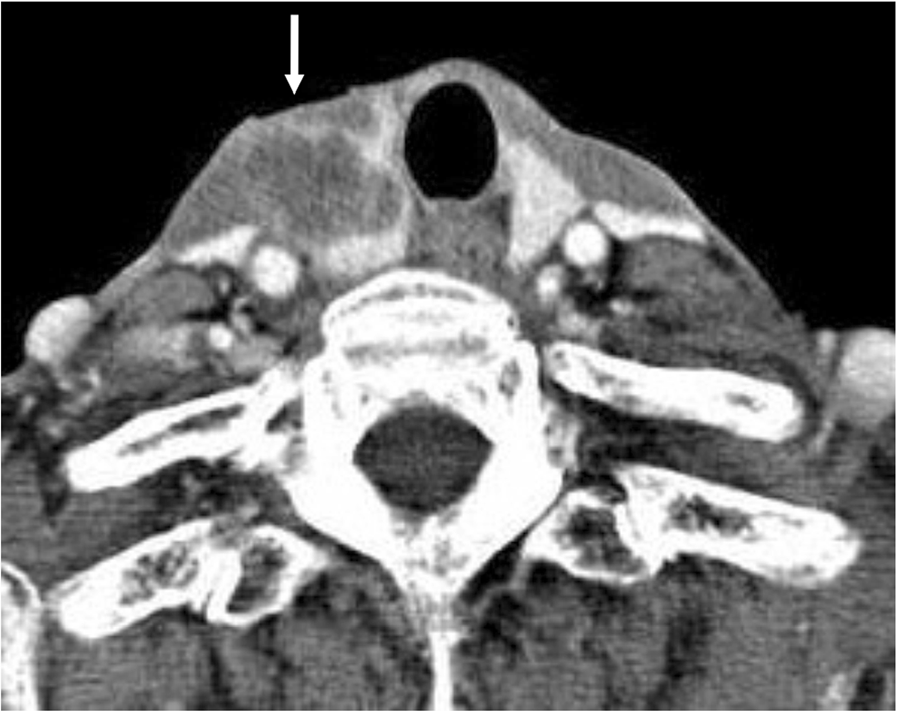
Computed tomography of mass in the thyroid gland. The invasion of muscles in the anterior region is indicated with an arrow

Computed tomography (CT) revealed a mass in the thyroid gland invading the muscles in the anterior region of the neck (Fig. 1). Paratracheal lymph nodes metastasis was also detected. However, no lateral cervical lymph node metastasis or distant metastasis was detected on CT and positron emission tomography. On the basis of cytology findings, poorly differentiated carcinoma was suspected. Additional systematic laboratory tests indicated normal thyroid function (thyroid-stimulating hormone [TSH] 4.0 IU/mL, F-T4 0.77 ng/dL), calcium levels (9.8 mg/dL), carcinoembryonic antigen (CEA) levels (0.5 ng/mL), and calcitonin levels (25 pg/mL); none of the findings indicated medullary carcinoma. However, the thyroglobulin (TG) level was slightly elevated at 98.4 ng/mL (normal range: <30 ng/mL). A poorly differentiated thyroid carcinoma was diagnosed, and hemithyroidectomy with central cervical lymphadenectomy was performed.

**Fig. 2:**
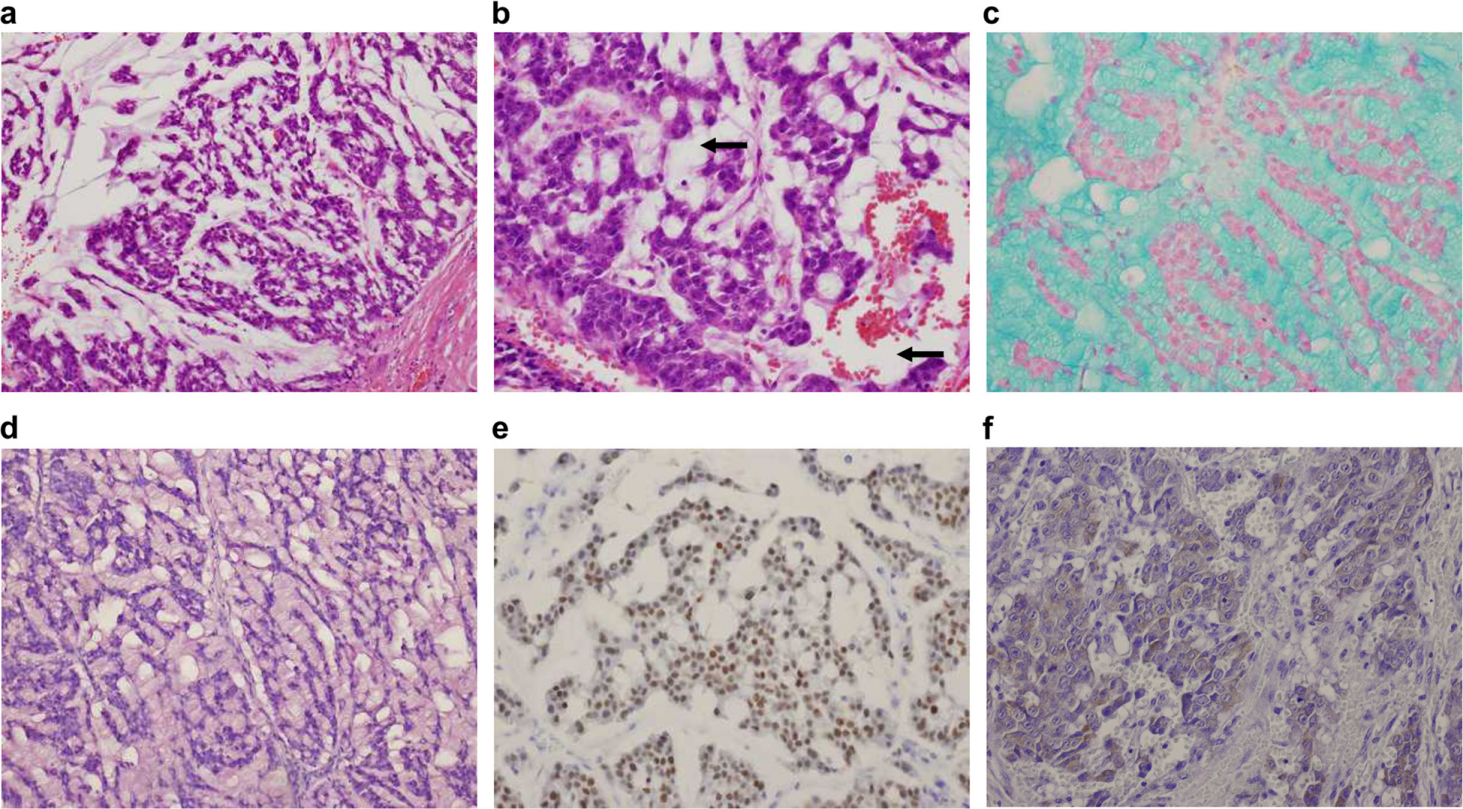
Pathological and immunohistochemical staining resulting in the diagnosis of primary mucinous carcinoma. **a** A large pool of extracellular mucin (hematoxylin-eosin, 10×). **b** Abundant mucus with large atypical cells (hematoxylin-eosin, 40×; the arrows point to the extracellular mucin). **c**: Mucinous material positive with Alcian blue staining. **d** Mucinous material positive with mucicarmine staining. **e** Tumor cells immunoreactive for thyroid transcription factor-1 (immunoperoxidase, 10×). **f** Tumor cells immunoreactive for thyroglobulin (immunoperoxidase, 40×)

**Fig. 3:**
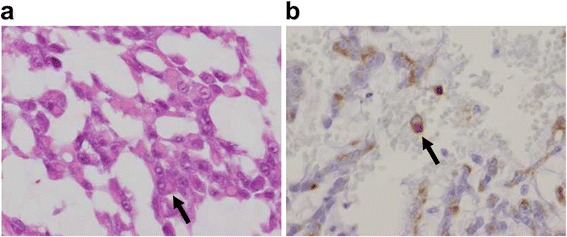
Pathological and immunohistochemical staining for the detection of rhabdoid cells. **a** Rhabdoid cells (hematoxylin-eosin 40×, arrow). **b** Rhabdoid cells positive for vimentin (arrow)

Pathological examination of the surgical specimen revealed abundant mucus as a characteristic finding (Fig. 2a). Agglomerations of large atypical cells were also detected (Fig. 2b). The large tumor cells had large atypical nuclei and a relatively high frequency of mitotic figures. Of note, rhabdoid cells with cytoplasmic inclusion bodies were detected scattered among the tumor cells. There were no nuclear grooves or intranuclear inclusions characteristic of papillary carcinoma, no papillary or follicular structure, and no necrosis or calcification. The extracellular mucus stained diffusely with Alcian blue staining (Fig. 2c), mucicarmine staining (Fig. 2d), and periodic acid–Schiff (PAS) staining, and these findings confirmed the epithelial origin of the mucin. In addition, the tumor cells stained positive for AE1/AE3, confirming the epithelial nature of the tumor. Thyroid transcription factor-1 (TTF-1) and TG staining was also positive, confirming the tumor’s ability to differentiate into thyroid tissue (Fig. 2e, f). To confirm whether the tumor was a medullary carcinoma, staining for calcitonin and CEA was also performed, but the results were negative. MIB-1 expression was moderately positive (40 %). The cytoplasmic inclusion bodies of some of the tumor cells were positive for vimentin, indicating that the cells were rhabdoid cells (Fig. 3a, b). On the basis of the above-mentioned findings, a primary mucinous carcinoma of the thyroid gland with rhabdoid cells was diagnosed.

**Fig. 4:**
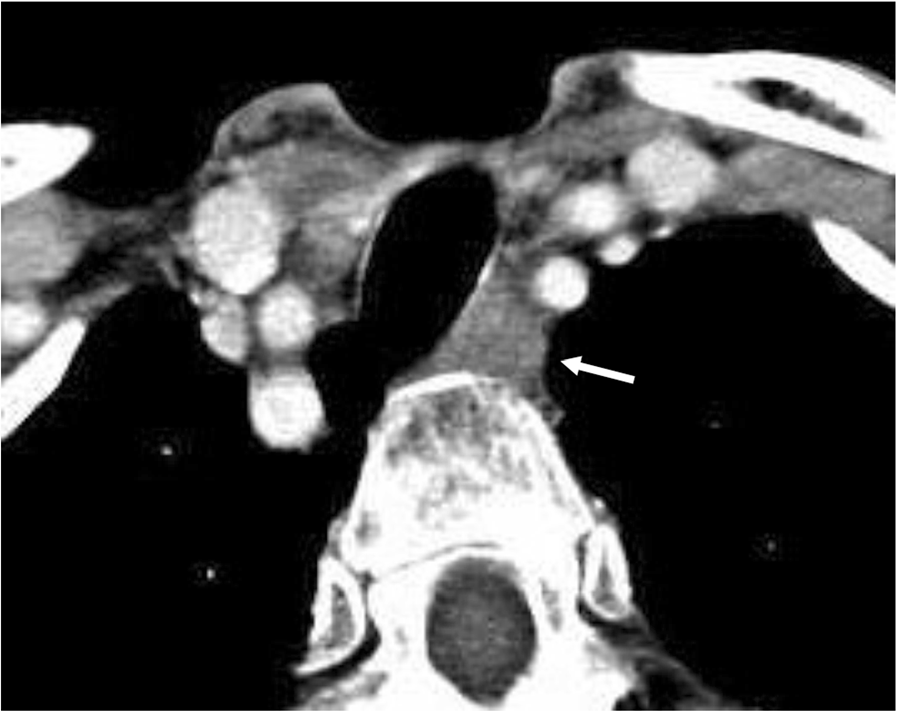
Computed tomography indicating recurrence in the paratracheal lymph node. The paratracheal lymph node is indicated with an arrow

Ten months after the surgery, recurrence was detected in the contralateral paratracheal lymph node (Fig. 4). Accordingly, total resection of the residual thyroid gland and paratracheal lymphadenectomy were performed. TSH suppression therapy was initiated as adjuvant therapy. Six years after treatment for the relapse, the patient is alive and disease-free. The TG levels decreased to <5.0 ng/mL after the surgery and have not increased subsequently, not even at the time of the relapse.

## Conclusions

Mucinous carcinomas are cancers characterized by an abundant extracellular production of mucus. A definitive diagnosis of primary mucinous carcinoma of the thyroid gland is difficult to establish, as some types of papillary carcinomas, follicular carcinomas, medullary carcinomas, and thyroid metastases from cancers of other organs also produce mucin, which is an important feature of primary mucinous carcinomas of the thyroid gland [5–7]. Various tests, including immunostaining, need to be performed to establish a pathological diagnosis of mucinous carcinoma and to confirm that the mucin in extracellular mucus is produced by the epithelium [4]. In the case described here, 3 staining methods, namely Alcian blue, mucicarmine, and PAS staining, were used to confirm the production of mucin in the mucus by the epithelium. Next, other histological types of mucus-producing tumors were ruled out. For example, papillary carcinoma and follicular carcinoma were ruled out based on the absence of follicular and papillary structures, the absence of hyaline nuclei or nuclear grooves, and the absence of intranuclear cytoplasmic inclusion bodies. Medullary carcinoma was ruled out based on the absence of amyloid deposits in the interstitial space between the cell aggregates and negative immunostaining for CEA and calcitonin. Also, positive TG and TTF-1 staining confirmed that the tumor originated from the thyroid gland and had the ability to differentiate into thyroid tissue, thus ruling out the possibility of a thyroid metastasis from a mucinous carcinoma of another organ. Finally, the tumor cells showed positive AE1/AE3 staining, providing further evidence of the epithelial nature of the tumor. Clinically, a primary mucinous carcinoma of the thyroid gland was diagnosed because no other primary tumor was detected on a whole-body scan, and similar tumor metastases were detected at the central cervical lymph nodes.

The characteristics of the 6 previously reported cases as well as the case described here are presented in Table 1. Lymph node metastasis was detected at the initial diagnosis in a high proportion of cases (6 of 7 cases). Furthermore, distant metastases developed in 3 out of 7 cases, and most patients died of the disease (5 of 7 cases); those who underwent incomplete resection died within a few months, demonstrating a poor prognosis [3–8]. Therapeutic methods for recurrence included surgery, iodine therapy, radiation therapy, and anticancer drug therapy, but none of these were reported to be effective [4–6]. Therefore, complete resection during the initial treatment appears to be the most effective treatment option.

**Table 1:** Summary of seven case studies with primary mucinous carcinoma

First author (year)	Age/sex	LN/distant metastasis	Treatment	Prognosis
Diaz [3] (1976)	44/M	No/No	Hemithyroidectomy immediately after diagnosis, followed by total thyroidectomy + ND	7y NED
Sobrinho [4] (1986)	56/M	Yes/No	Total thyroidectomy + ND	1y Recurrence (intestinal tract and lungs) **Tx**: RT and CH (5FU) 2y DOD
Cruz [5] (1991)	32/F	Yes/No	Total thyroidectomy + ND	2 m Recurrence (thyroid gland, skin, and lungs) **Tx**: iodine therapy, RT, CH (BLM, VCR, ADM) 8 m DOD
Kondo [6] (2005)	82/F	Yes/No	Hemithyroidectomy + ND	2y Recurrence (LN and skin) **Tx**: surgery and iodine therapy 4y DOD
Antonio [7] (2007)	62/F	Yes/No	Total thyroidectomy + ND (incomplete resection)	6 m DOD
Mnif [8] (2013)	56/M	Yes/No	Total thyroidectomy + ND (incomplete resection)	1 m DOD
Present case	81/F	Yes/No	Hemithyroidectomy	10 m Recurrence(LN) **Tx**: Total thyroidectomy + TSH suppression therapy 6y NED

*M* male, *F* female, *LN* lymph node, *ND* neck dissection, *NED* no evidence of disease, *DOD* died of disease, *RT* radiotherapy, *CH* chemotherapy, *5FU* 5-fluorouracil, *Tx* treatment, *BLM* bleomycin, *VCR* vincristine, *ADM* adriamycin

In the case reported here, additional surgery for ablation of the residual thyroid gland and prophylactic cervical lymphadenectomy were not performed initially because of the patient’s refusal to undergo these procedures. However, local recurrence developed 10 months later, and complete resection of the residual thyroid gland with TSH suppression was performed; no recurrence has been noted for 6 years. TSH suppression was continued, as the tumor cells had a tendency to differentiate into TG-producing cells and could express TSH receptors, as evidenced by positive TG staining and a decrease in TG levels to <5.0 ng/mL after tumor resection. Although the extent of the contribution of TSH suppression therapy to the prognosis in the case presented here remains unclear, the long-term survival suggests that TSH suppression therapy may be a suitable option for postoperative adjuvant therapy for mucinous carcinoma in selected cases.

Rhabdoid cells are large polygonal cells that contain an intracellular eosinophilic hyaline substance, and these cells are immunohistochemically positive for vimentin and keratin. These cells have been reported to be detected in various types of malignant tumors. It has been suggested that rhabdoid cells represent a stage of cellular degeneration or a preliminary stage before apoptosis or cell necrosis [9]. There are also few reports on the presence of these cells in thyroid cancer. In all cases, the presence of rhabdoid cells has been reported as an indicator of poor prognosis [10–12].

Histologically, the case described here was also predicted to have a poor prognosis; however, long-term survival of 6 years has been achieved. We encountered a rare case of primary mucinous carcinoma of the thyroid gland; the presence of rhabdoid cells, which are rarely found in thyroid carcinomas, was also observed. To our knowledge, this is the first report to describe the presence or rhabdoid cells in a primary mucinous carcinoma of the thyroid. Histopathologically, this case is extremely rare, and clinically, long-term survival in such cases is also very rare, but this was achieved with complete tumor resection and TSH suppression therapy in this case. The pathological diagnosis of primary mucinous carcinoma of the thyroid gland is difficult to determine and requires thorough histopathological analyses. In addition, prompt and complete resection of the tumor immediately after the diagnosis may be the most effective treatment strategy.

## Abbreviations

CEA, Carcinoembryonic antigen; CT, Computed tomography; PAS staining, Periodic acid–Schiff (PAS); TG, Thyroglobulin; TSH, Thyroid-stimulating hormone; TTF-1, Thyroid transcription factor-1
